# Editors’ Book Table

**Published:** 1874-08

**Authors:** 


### Editors' Book Table.

NOTE. — All works reviewed in the columns of the CHICAGO MEDICAL JOURNAL may be found in the extensive stock of W. B. KEEN, COOKE & CO., whose catalogue of Medical Books will be sent to any address upon request.

*A Conspectus of the Medical Sciences, Comprising Manuals of Anatomy, Physiology, Chemistry, Materia Medica, Practice of Medicine, Surgery and Obstetrics, for the use of Students.* By HENRY HARTSHORNE, A.M., M.D., Professor of Hygiene in the University of Pennsylvania, etc., etc. Second Edition. Enlarged and thoroughly revised, with four hundred and seventy-seven illustrations. Philadelphia: Henry C. Lea.

This is perhaps the best book of its class which has hitherto appeared; it is full, complete and well arranged. Designed for the use of students, it is, however, liable to abuse by this class of readers, in supplying to them a ready method of cramming for examinations. This fact constitutes the basis of our objection to this entire class of manuals, and with this fact in view our endorsement of the book must be qualified thereby. H.

*An Introduction to Physical Measurements, with Appendices on Absolute Electrical Measurements, etc.* By Dr. F. KOHLRAUSCH, Professor-in-Ordinary at the Grand-Ducal Polytechnic School at Darmstadt, and formerly Professor of Physics at the University of Gottingen. Translated from the Second German Edition, by THOMAS HUTCHINSON WALLER, B.A., B.Sc., and HENRY RICHARDSON PROCTOR, F.C.S. New York: D. Appleton & Son. 1874.

The scientific physicist or chemist will find in this little treatise an invaluable hand-book for the laboratory, and while there is much that is both valuable and interesting to the physician, using the term in its ordinarily restricted sense, its thorough appreciation demands a degree of proficiency in mathematical science which is acquired by very few medical men. Indeed, while the processes for the determination of physical measurements are most of them examples of conscientious labor and models of scientific accuracy, they concern matters too minute, and involve investigations too delicate, to come within the scope of the practical physician in his daily routine of duties. But of this class, even, those who have the time to devote to it will derive much valuable instruction from its pages. H.

*Electro-Therapeutics: A Condensed Manual of Medical Electricity.* By D. F. LINCOLN, M.D., Physician to the Department of Diseases of the Nervous System, Boston Dispensary. Philadelphia: Henry C. Lea. 1874.

One of the best manuals of electro-therapeutics hitherto published. It is an effort in the right direction, to reduce order out of chaos, and to deduce some general principles of application from the heterogeneous mass of empiricism commonly denominated electro-therapeutics. The work is comprised in eight chapters: Physical Laws of Electricity; Modes of Generating It; Physiology; Diagnosis; Modes of Application; Medical and Surgical Practice; Cautions; and Apparatus. They will be found comprehensive, and yet concise, and bear the impress of a mind practically familiar with his subject. H.

NEW JOURNALS.

The present season has thus far been prolific in additions to the periodical professional literature. Amongst these *The Archives of Electrology and Neurology* occupies a conspicuous position, not only from the novelty of the field which it proposes to occupy, being the first legitimate effort in that direction in America : but likewise from the deservedly eminent position held by its Editor, Dr. Geo. M. Beard, in this department of scientific inquiry. Under the editorship of so able an investigator, and so accomplished a teacher as Dr. Beard, we have the right to expect a journal of a high order of merit, which will become the medium for the presentation to the profession of what is true and reliable, and the exposure of what is fallacious in this terra incognita of electrology. The literature of electricity, more especially in its therapeutical relations, is becoming voluminous, although by no means proportionately perspicacious. To digest this mass will become one of the most important functions of a journal such as this, in the performance of which it will render a signal service to the reading portion of the profession. The hopelessness which has hitherto oppressed the minds of practical men, in regard to the successful treatment of many forms of nervous disease, in view of the brilliant successes attributed to electrical influence in some of these forms, has given place to a spirit of credulity which demands both direction and repression, and this, too, will become one of the duties of Dr. Beard's Archives. The present No. justifies our expectations; it is creditable alike to both editor and publisher. It would give us pleasure, did time and space permit, to notice its contents in detail. We shall take the liberty hereafter to draw freely upon them for the benefit of those of our readers who may not be so fortunate as to become its subscribers.

H.

In the *Psychological and Medico-Legal Journal*, we prefer to claim an old friend returned to the work after a short absence, in a somewhat modified garb, than an entirely new acquaintance. It was with great regret that we chronicled the cessation of the publication of Dr. Hammond's Journal of Psychological Medicine, from whose pages we have drawn many lessons both pleasant and profitable, and it is with much pleasure that we welcome the advent of its legitimate successor to a field of professional literature largely unoccupied. In this field, the new journal will, of course, from the established reputation of its editor as an authority in psychological medicine, assume the most conspicuous position, and will be gladly welcomed by all students in this department of medical science. The new is somewhat more comprehensive in its scope than the old journal was, since it is intended to include the department of Medical Jurisprudence, which is by no means as attentively regarded by the majority of the profession as it deserves. The present issue is designed to be a monthly instead of a quarterly as in the former case, which change in form is asserted by the editor to be in opposition to his own judgment, and only a concession to that of his friends. It contains the address of the editor, upon assuming the presidency of the New York Neurological Society, upon the "Effect of Alcohol upon the Nervous System;" the Laws of Topographical Diagnosis, in Chronic Diseases of the Nervous System, by Moritz Benedict; the Proceedings of the N. Y. Neurological Society; and Reviews; all of which will be found interesting. The Editor is assisted by Dr. T. B. Cross. The Publisher, F. W. Christern, 77 University Place. The local agents, Messrs. W. B. Keen, Cooke & Co.

Another new candidate for periodical honors appears from the South, in the shape of "*The American Medical Weekly*," edited by Dr. E. S. Gaillard, of Louisville, Ky. Dr. G., so long and favorably known to the medical profession as the editor of the *Richmond and Louisville Medical and Surgical Journal*, one of the largest and best of our cotemporaries, must be endowed with much more than his fair share of energy and industry, who can, while already conducting a medical journal of no mean pretensions, thus undertake the conduct of another; who in no dread of monthly, braves the terrors of weekly visitations of the Devil (Printers.) We can only wish for ourselves that his industry were transmissible through the mails. We can pay both journals no higher compliment than the Horation "Mater pulchra filia pulchrior." H.

From the antipodes comes the *Australasian Medical and Surgical Review*, giving us a peep at the sayings and doings of our professional brethren in "isles of the sea." The No. before us, the 7th, vol. 1, contains interesting original articles upon Leucorrhea, Diarrhea, and Anæsthetics, showing the profession to be well up to the times in the antarctic world; Extracts, and Items; from the latter of which which we are glad to learn the Christian spirit pervading the profession, as demonstrated by the omission of the meeting of the Victoria Medical Board on Good Friday—an example worthy of imitation.

Its columns contain also the results of the Examination at the University of Melbourne, from which it appears that the course of medical studies extends over a period of five years—another good example, still more worthy of imitation. H.

BOOKS RECEIVED.

*Inflammation of the Lungs, Tuberculosis and Consumption.* By LUDWIG BUHL. New York: G. P. Putnam's Sons, Fourth avenue and Twenty-third street.

*Electro-Therapeutics; A Condensed Manual of Medical Electricity.* By D. F. LINCOLN, M.D., Physician to the Department of Diseases of the Nervous System, Boston Dispensary. Philadelphia: Henry C. Lea. 1874.

PAMPHLETS RECEIVED.

*Catalogue of the Officers and Students of the University of Virginia.* Fiftieth Session, 1873–74.

*Fifteenth Annual Announcement of the Miami Medical College of Cincinnati.* Session of 1847-5.

*Electrolysis in the Treatment of Stricture of the Urethra.* By ROBERT NEWMAN, M.D., New York.

*Thirty-third Annual Announcement of the St. Louis Medical College.* Winter Session, 1874–5; and Catalogue for 1873–4.

*Transactions of the Minnesota State Medical Society*, 1874. St. Paul.

*Medical Problems of the Day.* The Annual Discourse before the Massachusetts Medical Society, June 3, 1874. By NATHAN ALLEN, M.D., LL.D., Lowell, Mass.

*Transactions of the Kentucky State Medical Society*, 1874.

*Inorganic Cardiac Murmurs.* By A. T. KEYT, M.D., Cincinnati.

*Fifty-fourth Annual Announcement of the Medical College of Ohio.*

*Report of the Quebec Lunatic Asylum*, 1872–3.

*Twenty-fifth Annual Announcement of the Medical Department of the University of Nashville.*

*Diseases of the Conjunctiva.* By DUDLEY S. REYNOLDS, M.D., Professor of Ophthalmology and Otology in Louisville Hospital Medical College, Louisville, 1874.

JOURNALS RECEIVED.

The Atlanta Medical and Surgical Journal—July.
" Australasian Medical and Surgical Journal, Melbourne—April.
" American Journal of the Medical Sciences—July.
" American Practitioner, Louisville—July.
" American Medical Weekly—Vol. 1, No. 2.
Boston Journal of Chemistry—July, and Index.
Boston Medical and Surgical Journal.
The Clinic, Cincinnati—June 20, 24, July 11.
Cincinnati Lancet and Observer—July.
Druggists' Circular—July.
Detroit Review of Medicine and Pharmacy—July.
Le Gazette Medicale de Paris—No. 21.
The Indiana Journal of Medicine—June, 1874.
London Lancet—June, 1874.
The Medical Times, Philadelphia—June 20, 27, July 1.
" Medical and Surgical Reporter, Philadelphia—June 20, 27, July 4, 11.
" Medical Examiner, Chicago—June 15, July 1.
" Medical Record, New York—June 15, July 1.
" Missouri Clinical Record—July.
" Medical Press and Circular, London—June.
" Medical Mirror, New York—July.
" Medical News and Library—July, and Supplement.
" Medical Herald, Leavenworth—July.
" Northwestern Medical and Surgical Journal—July.
" Nashville Journal of Medicine and Surgery—June.
" Peninsular Journal, Detroit—March and July, 1874.
" Pharmacist, Chicago—June, 1874.
Le Progres Medicale—March 23, 30, June 6, 13.
The Practitioner, London.
" Physician and Pharmacist—May.
La Reforma Medica Periodico Oficial, Madrid.
The Richmond and Louisville Medical Journal—June.
" St. Louis Medical and Surgical Journal—July.
" Southern Medical Record—June.
" Virginia Medical Monthly—July.

